# Burden of asthma in the hospital setting: an Australian analysis

**DOI:** 10.1111/j.1742-1241.2007.01559.x

**Published:** 2007-11

**Authors:** L. Watson, F. Turk, K. F. Rabe

**Affiliations:** 1 Louise Watson Consulting Ltd Buxton, UK; 2 Novartis Pharma AG, Lichtstrasse Basel, Switzerland; 3 Department of Pulmonology C3-P, Leiden University Medical Center Leiden, The Netherlands

## Abstract

**Objectives:**

This study was designed to report factors associated with asthma hospital admission, such as patient characteristics, type of admission and subsequent outcome i.e. discharge or death, for the years 2000–2005. These data are used for health economic models regarding asthma burden in the hospital setting in Australia.

**Methods:**

Data was obtained from the Australian Centre for Asthma Monitoring using their amalgamated dataset from all states and territories. Admissions under ICD-10 codes J45 ‘Asthma’ plus all subcodes, and J46 ‘acute severe asthma’ were included. Codes for associated comorbidity at the time of admission were identified, as well as the month of death, age, gender and length and the type of stay. Confidence intervals for death rate assumed a binomial distribution because of the rarity of event.

**Results:**

The total number of all-cause deaths for the 5-year observation period was 289 from 202,739 asthma separations or 0.14% or 143 deaths/100,000 separations and the highest rate was seen in patients over 45 years. Acute upper respiratory tract infections were reported in up to 25% of all asthma hospital admissions. Length of stay was up to a mean average of 10.2 days in patients who died (SD 15.3). In 5 years observation there was 152,758 emergency asthma admissions which contributed greatly to Australian healthcare burden.

**Conclusions:**

The study demonstrates that emergency admissions dominate asthma care in the hospital setting in Australia, which suggests poor asthma control in some patients with subsequent economic burden. Asthma-related mortality remains a risk for specific patients in the hospital setting.

**What's known**

To date Australian asthma fatality data has been reported by ACAM, but not specifically with the idea of comparison with UK or US studies run with similar methodologies, and with the purpose of identifying areas of highest burden within the hospital systems and the risk factors associated with mortality in asthma patients.

**What's new**

This study provides the additional data for international comparison as it evaluates the admission type and asthma disease burden by ICD code, and describes the population both admitted and at risk of all-cause mortality.

## Introduction

Asthma is a leading cause of morbidity and can often lead to hospitalisation and in some severe cases, death. Asthma burden has both health system and social costs. Clearly, in countries with a high prevalence of asthma, health resource utilisation will be greater than countries with lower prevalence. The Australian Bureau of Statistics estimates that asthma prevalence in 2004–2005 was 10% (2 million people) using the National Health Survey (1). Australia has previously been reported as having the second highest prevalence of self-reported wheeze in adults 20–44 years of 41 countries surveyed in the extended European Community Respiratory Health Survey (2). Kenny et al. (3) reported that in a prospective, longitudinal study of 245 asthma patients aged 5–75, the greatest costs to the health system were driven by admitted hospital care. The minority of patients who required such treatment, substantially skewed the cost distribution as the majority of patients only utilised general practice treatment. In a cost-effective evaluation in Australia, Simonella et al. (4) calculated that optimal asthma treatment and compliance would avert the need for interventions such as hospitalisation, with a 69% saving of the current cost burden.

This analysis of the asthma admissions and mortality rates within the Australian hospital system for the years 2000–2001 to 2004–2005 inclusive gains greater insight into current asthma burden and provides a better understanding of the asthma population requiring hospital treatment. The population of admitted patients is described by age, gender and comorbidities. Length of stay and season of death are also reported.

## Methods

Data was taken from the Australian Institute of Health and Welfare database run in collaboration with the Australian Centre for Asthma Monitoring, a group set up to assist in reducing the burden of asthma in Australia by developing, collating and interpreting data relevant to asthma prevention, management and health policy. The database contains the asthma public and private hospital inpatient information from all Australian states and territories. Data in this study runs from 1st July 2000 until 30th June 2005. In Australia hospital events are reported as ‘separations’. Rates are presented per number of separations and may not correspond to actual patient numbers as patients may have more than one separation per episode or admission period. Data was not available at the patient level.

Australia uses ICD-10 coding where J45 codes as J45.0-allergic asthma, J45.1-non-allergic asthma; J45.8-mixed asthma, J45.9-asthma unspecified and J46 refers to acute severe asthma ‘status asthmaticus’. Coding is applied by a medical coder after patient discharge. Medical coders are not physicians and merely apply codes based on the information contained within the medical record. However, as it is not known how rigorously the codes are applied, it was decided to limit reporting here to J45 codes combined and J46 codes respectively. It is thought that the application of these codes is reasonable, although clearly it cannot be guaranteed as to their 100% accuracy. This is a limit of any database used for epidemiological analysis. In the database J45 and J46 codes are also divided into elective and emergency events. Emergency does not necessarily correspond to an admission via the emergency department but the term refers to an event where care or treatment, in the opinion of the treating physician, must occur within 24 h of examination i.e. as a matter of relative urgency. Where the number of separations is ≤ 5, the number is not reported because of confidentiality rules in place within the Australian data protection system, as it is believed that individuals may be able to be identified from such small numbers. Ethical permission was not required for this study as the data is anonymised and the patient identification is impossible in the way it is aggregated.

Results are reported by age band where appropriate, namely; 0–11; 12–16, 17–44 and ≥ 45. These age bands were selected based on common prescribing banding for asthma medications and to also categorise patients by age groups that have differing mortality and morbidity risks in the general population, as it is important to apply such risk criteria to the clinical population. We did not attempt to further divide the older age band as number of events such as mortality are very low within each strata and it was not felt appropriate to categorise further.

No exclusion criteria were applied to the selection of data, to reflect the real world scenario. Mortality that occurred during the admission spell (the period from a live admission to either death or discharge) were reported. Death is for all cause as it is not possible to obtain the death certificate to verify the cause of death from this database. We were not able to assess medication use in this study, as the pharmacy records of the patients were not held as part of the admission or discharge records. We are therefore not able to ascertain if cause of death was in any way related to care or treatment, either prior to, or during the hospital episode. We were additionally not able to assess smoking or allergy status as these are not recorded in the database. Separations, mortality, length of stay, presenting comorbidity and the month of death were reported, stratified by age and gender where appropriate.

The analyses took the form of descriptive statistics. Confidence intervals for the death rates post asthma admission were created assuming a binomial distribution using the ‘Exact’ method (5). Binomial distribution is considered more appropriate than a normal distribution when the data is skewed and there are only a few cases. The number of deaths per number of separations was converted into deaths per 100,000 separations. Analyses were conducted using STATA V9.2 (Stata Corporation, College Station, TX, USA).

## Results

**Figure 1:**
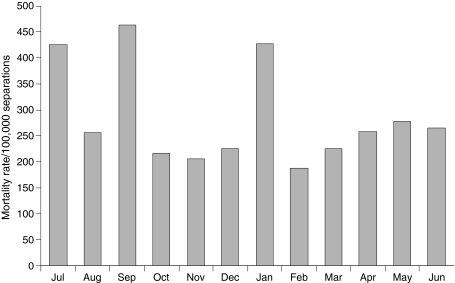
Month of death for adult asthma patients who died postadmission 2000–2001 to 2004–2005 (the rate is taken as the mean average across the 5-year observation period)

The numbers of asthma hospital events (separations) are shown in Table 1 by type of admission. It can be seen that for all codes, emergency (care within 24 h) events are by far the most common. The total number of deaths over the 5-year period for all codes combined equalled 289 from 202,739 asthma hospital separations or 0.14%. Table 2 shows the mortality rate per 100,000 separations and the data indicate that when considered as a rate, males have a higher rate in the 17–44 age group than females, particularly so in the J46-coded patients. This is not observed in the older patients over 45 years of age. For the assessment of mortality throughout the year only adult data were used as paediatric death was considered too rare to assess any monthly patterns. Figure 1 illustrates that July, September and January have the highest rates.

**Table 2:** Mortality rate per 100,000 separations by gender and age group 2001–2001 to 2004–2005 inclusive

	J46		J45	
		
Age band (years)	Male rate (95% Cl)	Female rate (95% Cl)	Male rate (95% Cl)	Female rate (95% Cl)
0–11	n.p.	n.p.	n.p.	n.p.
12–16	n.p.	n.p.	0	0
17–44	900 (571; 1347)	351 (211; 547)	80 (38; 147)	44 (22; 79)
45 and over	1178 (748; 1763)	1116 (839; 1454)	318 (222; 442)	329 (266; 401)

n.p., numbers < 5 so data not presented.

**Table 1:** Acute admissions by asthma code, 2000–2001 to 2004–2005 inclusive

	Day case			Not day case		
		
	Elective	Emergency	Unknown	Elective	Emergency	Unknown
J46	101	4926	18	1724	30,316	406
J45	1808	29,882	201	8994	122,442	1788
Total	1909	34,808	219	10,718	152,758	2194

The secondary comorbidities of patients admitted with asthma are shown in Table 3. For all admissions, acute upper respiratory infections were the most common comorbidity. Length of stay for those patients who died and those who were subsequently discharged are shown in Table 4. Mean data are provided to inform health economics as total resource use must be documented and the mean is the only method suitable for measuring the average for total values. Patients who died had longer average stays than those who were discharged.

**Table 4:** Length of stay for patients admitted with asthma between 2000–2001 and 2004–2005

	Mean	SD	Median	IQR
*Length of stay for patients who died post asthma admission*				
J46	8.6	15.1	3	5.5
J45	10.8	15.3	5	10
*Length of stay for patients discharged post asthma admission*				
J46	3.1	4.9	2	3
J45	2.7	3.3	2	2

SD, standard deviation; IQR, interquartile range.

**Table 3:** Secondary comorbidities listed in 3% or more of patients admitted with asthma in the year 2004–2005

Secondary comorbidity code	Asthma admission code	Number of patients with comorbidity	% of patients comorbidity	Asthma admission code	Number of patients with comorbidity	% of patients comorbidity
J06 acute upper respiratory infections	J46	905	17.2	J45	8132	25.3
Z71 medical advice or counselling	J46	715	13.6	J45	0	0
Z72 problems related to lifestyle	J46	460	8.7	J45	3103	9.6
Z86 personal history of certain other diseases	J46	257	4.9	J45	1751	5.4
110 essential primary hypertension	J46	195	3.7	J45	1355	4.2
J22 unspecified acute lowerrespiratory tract infection	J46	188	3.6	J45	1712	5.3
E11 type 2 diabetes	J46	188	3.6	J45	1484	4.6
J96 respiratory failure	J46	162	3.1	J45	0	0

## Discussion

This database analysis shows that mortality in asthma patients after a hospital admission occurs predominantly in older patients over 45 years of age. However, the principle area of asthma burden to the health system lies in the emergency or urgent admissions requiring care within 24 h, which occurred in large numbers, relative to all admissions for asthma throughout Australia during the 5-year observation period. A weakness of any database analysis is the reliance on aggregated coded data, where coding reliability is not validated. For example, it is perhaps surprising to see that some J46 status asthmaticus events were additionally coded as elective i.e. not an emergency. We cannot check if this is an error or the correct coding of patients admitted with status asthmatics but not considered to require emergency admission by the attending physician. However, we believe that this database is no more or less reliable than any other and additionally that it provides a reasonable overview of asthma admissions within Australia and the subsequent burden to the health system.

The majority of these were inpatient stays and due to some patients with very long stay, the mean stay was up to 10 days in those patients who subsequently died, and 3 days in those who were discharged. This clearly has high cost impact on the health system and is supported by Kenny et al. (3) who reported that in Australia, most of the health system burden consisted of inpatient hospital care. Kenny also reported that the costs attributed to asthma were skewed by some individuals who had very high per capita costs, the distribution thus ranging from AUS $0 to $4882 per annum. Whilst this study only reports at the event level rather than patient level because of data aggregation, it is likely that a proportion of patients will have repeated attendance at the hospital and contribute greatly to cost. This has been found in other studies about emergency asthma admissions and fatal asthma, where a risk factor for death or emergency admission were prior admissions (6,7). Why patients may repeatedly require admission is not assessed here, but has previously been suggested in the TENOR study to be due to poor control in moderate-to-severe asthma patients (8). The TENOR study reported that severe asthma patients have the highest healthcare utilisation with 5% of adults and 10% of children having experienced hospitalisation in the last 3 months and up to 13% having required intubation. Vollmer et al. (9) also reported that acute healthcare utilisation was three times more likely when patients had control problems. The findings in this study also support Simonella's assertion that an optimal treatment model with at an investment of AUS $627 million would avert 69% of the disease burden (4). Whilst this model costed investment at 39% more than the current cost of treatment, it was offset by a greater than twofold increase in health benefit, with emergency department and hospital separations estimated to decrease by 28%. Optimal treatment would also include general practitioner (GP) adherence to guidelines and re-identification of GPs priorities for delivery of asthma care, which has been reported to be discordant in Australia (10).

This study reports a slightly lower mortality rate than a recent study examining UK mortality post asthma admission, at 0.14% of all asthma admissions vs. 0.43% in the UK (11). A US study also reported in-hospital mortality post asthma admission at 0.5% (12). Thus, whilst the majority of Australian asthma patients are successfully managed in hospital, some still die. Mortality may have many contributory factors including accessibility to care, social status or ethnicity, severity, triggers, treatment and so on, which have been shown in other studies to impact on mortality rates (7,12,13). Those patients that died were mainly from the older age group aged over 45 years, a finding supported by the UK and US studies which also found mortality to be far higher in the older patients (11,12). Comorbidities experienced by these patients may have contributed to death or certainly caused the asthma exacerbation. Upper respiratory tract infection, experienced in this study by up to 25% of patients, has been previously related to near fatal asthma and exacerbations (14). Ten Brinke et al. (13) found that comorbidities contributed to exacerbations in 136 severe asthma patients, particularly repeated respiratory tract infections, which was the second highest risk for frequent excerbations [OR 6.9 (95% CI: 1.9–24.7)], behind psychological dysfunctioning [OR 10.8 (95% CI: 1.1–108.4)], which may have been caused by the asthma rather than contributing to the exacerbation rate.

Slightly more deaths occurred in the Australian winter months (June through to September) with another small peak in January (mid-summer). There is no clear pattern, however, for asthma deaths as observed elsewhere and it is hard to relate them to cause such as the respiratory virus seasons which is more clearly observable in the northern hemisphere (11).

This study has shown that in Australia the major burden to the hospital system from asthma is emergency hospital admission and subsequent stay. Asthma-related mortality remains a risk for specific patients within the hospital setting. Mortality occurs mainly in older adults and the contributory effect of comorbidities suffered by these patients requires further investigation.
